# Scalable analysis of whole slide spatial proteomics with Harpy

**DOI:** 10.1093/bioinformatics/btag122

**Published:** 2026-03-13

**Authors:** Benjamin Rombaut, Arne Defauw, Frank Vernaillen, Julien Mortier, Evelien Van Hamme, Sofie Van Gassen, Ruth Seurinck, Yvan Saeys

**Affiliations:** Data Mining and Modelling for Biomedicine, VIB-UGent Center for Inflammation Research, 9000 Ghent, Belgium; Department of Mathematics, Computer Science and Statistics, Ghent University, 9000 Ghent, Belgium; VIB Center for AI and Computational Biology, 9000 Ghent, Belgium; VIB Spatial Catalyst, VIB Technologies, 9000 Ghent, Belgium; VIB Spatial Catalyst, VIB Technologies, 9000 Ghent, Belgium; VIB Spatial Catalyst, VIB Technologies, 9000 Ghent, Belgium; VIB Spatial Catalyst, VIB Technologies, 9000 Ghent, Belgium; Data Mining and Modelling for Biomedicine, VIB-UGent Center for Inflammation Research, 9000 Ghent, Belgium; Department of Mathematics, Computer Science and Statistics, Ghent University, 9000 Ghent, Belgium; VIB Center for AI and Computational Biology, 9000 Ghent, Belgium; Data Mining and Modelling for Biomedicine, VIB-UGent Center for Inflammation Research, 9000 Ghent, Belgium; Department of Mathematics, Computer Science and Statistics, Ghent University, 9000 Ghent, Belgium; VIB Center for AI and Computational Biology, 9000 Ghent, Belgium; Data Mining and Modelling for Biomedicine, VIB-UGent Center for Inflammation Research, 9000 Ghent, Belgium; Department of Mathematics, Computer Science and Statistics, Ghent University, 9000 Ghent, Belgium; VIB Center for AI and Computational Biology, 9000 Ghent, Belgium

## Abstract

**Motivation:**

Current spatial proteomics data analysis workflows are limited in efficiency and scalability when applied to gigapixel sized datasets. Moreover, they often lack extensive quality control tools and exhibit limited interoperability with existing spatial omics analysis ecosystems.

**Results:**

We introduce Harpy, a new Python workflow capable of accelerated processing of large spatial proteomics datasets. We demonstrate the utility of Harpy on four datasets and show that it can rapidly apply state-of-the-art segmentation and feature extraction via parallel processing. Each analysis step is accompanied by appropriate quality control steps. Scalable clustering of cells and pixels allows identification of cell types, processed up to 27 times faster than previously reported. Processing and visualization can be performed locally or on high-performance computing servers. Additionally, Harpy integrates well with existing spatial single-cell analysis tools in the Python and R software ecosystem.

**Availability and implementation:**

Harpy is available on GitHub at https://github.com/saeyslab/harpy and archived on Zenodo at https://doi.org/10.5281/zenodo.15546703.

## 1 Introduction

The advent of spatially resolved single-cell transcriptomics and proteomics has transformed our understanding of cell and tissue architecture (Bussi and Keren 2024, Carstens *et al.* 2024). These highly multiplexed bioimaging technologies have enabled the profiling of gene and protein expression in a spatial and subcellular context, providing insights into the molecular mechanisms underlying cellular function and tissue organization. Despite the significant promise of spatially resolved transcriptomics and proteomics, integrating and analyzing the resulting data remains complex. The speed of data acquisition has far outpaced the speed of available specialized computational tools and workflows capable of managing the substantial data volumes generated (Carstens *et al.* 2024). We differentiate between models, such as cell segmentation or cell clustering models, and software workflows applying those models to complete a complex data analysis task. While various models and workflows have been proposed for spatial proteomics (SP) and highly multiplexed protein imaging by immunofluorescence particularly, applying these models successfully to large whole slide imaging (WSI) data in a coherent workflow remains a hurdle. We identify three limitations of current state-of-the-art computational workflows that could be improved towards the future: quality control, scalable processing and interoperability. Scalable processing includes image segmentation, feature extraction and cell and pixel clustering.

### 1.1 Quality control and visual inspection

When analyzing SP data, performing rigorous quality control (QC) is advisable for every data processing step (Vierdag and Saka 2024). QC methods are usually designed for a specific data modality, such as Imaging Mass Cytometry (IMC) (Windhager *et al.* 2023) or CODEX tumor microarrays (Tan *et al.* 2024), although some methods can be agnostic to the data acquisition platform. These methods can range from qualitative visual inspection of images to more quantitative quality control metrics. Visual inspection is crucial, as statistics generated can poorly represent morphological information in images. At the same time, visual review must be backed up by objective methods that detect and correct human errors and biases (Baker *et al.* 2024). Specific difficulties for SP data are performing such quality control in a multi-sample context, as visually inspecting or calculating metrics over multiple whole slide samples for various markers of interest can be cumbersome, and taking into account metadata such as physical pixel size, exposure time, fluorophore, emission, or excitation wavelength. For example, access to the correct physical pixel size allows appropriate rescaling when applying cell segmentation models, improving speed and accuracy (Stringer *et al.* 2021). Some metadata, such as the order of marker acquisition, can be important for multicycle imaging technology platforms such as MACSima (Kinkhabwala *et al.* 2022) and Phenocycler (Black *et al.* 2021) but this is often not considered in any current tool.

### 1.2 Scalable processing of images and cells

SP datasets can be very large, as each protein is expressed as a raster of pixel values across the whole sample. This contrasts with spatial transcriptomics, where most acquisition platforms already summarize the biological signal to a point cloud of gene transcript locations. SP dataset sizes of original samples can range from giga-to-terabyte, hindering analysis as this size cannot easily be loaded into memory or sometimes not even be stored on the laptop of researchers. This makes many current computational workflows, like MCMICRO (Schapiro *et al.* 2022a) and ark-analysis (Liu *et al.* 2023), require large resources to fully process gigapixel scale images, hindering data analysis when access to expensive research infrastructure is limited. Methods like TRACERx-PHLEX (Magness *et al.* 2024), ark-analysis (Liu *et al.* 2022) and Fractal (Lüthi 2024) are designed primarily for modalities such as Imaging Mass Cytometry (IMC), Multiplexed Ion Beam Imaging by Time Of Flight (MIBI-TOF), and High-Content Screening (HCS) respectively, which typically have a low field-of-view compared to whole slide microscopy imaging, although whole slide imaging IMC has been recently reported (Kim *et al.* 2023). Most such tools have less need for parallelized WSI processing and can cope by only parallelizing batches of these small samples. While creating small region-of-interest (ROI) subsets from SP data can alleviate some processing issues, a data processing approach performed on the complete whole slide is free of region selection bias (Lee *et al.* 2024) and allows more seamless scaling when analyzing larger datasets (Rocklin 2015). We highlight four processing steps: image segmentation, feature extraction and cell and pixel clustering.

Accelerated deep learning segmentation model inference can be achieved by increasing inference speed using a GPU, but these can be prohibitively expensive, hard to set up and are usually even more limited in available working memory than the CPU. An out-of-core approach removes this limitation on available working memory and allows acceleration for any data size with either CPU or GPU. Larger-than-memory images are divided into data chunks, allowing each chunk to be used for parallel model inference. The result is the combination of the model output for each chunk. But depending on the implementation there is a chance of significantly affecting prediction quality and creating artifacts on the grid of tiles at the chunk borders (Buglakova *et al.* 2025). The parallel segmentation implementation in Squidpy (Palla *et al.* 2022) can have tiling artefacts in dense cell regions, which we will discuss further in the manuscript. Usually, most cell segmentation models like Cellpose (Stringer *et al.* 2021) offer a basic sliding-window implementation to process large datasets. However, applying different segmentation models to large biological images in a standardized fashion is still a time-consuming step for many popular workflows. Recent methods like Sopa (Blampey *et al.* 2024) tackle large images by splitting them into separate explicit tiles, processing them individually, and then merging the outputs into a single result. However, this approach quickly becomes complex, as it requires handling tile overlaps and postprocessing to correct border artifacts. Explicit tiling can also reduce performance when intermediate results must be serialized to disk or when task scheduling fails to consider optimal tile locality. Squidpy uses the popular Dask framework (Rocklin 2015) to simplify and optimize such distributed image processing. However, Squidpy does not fully support the new SpatialData format (Marconato *et al.* 2025), as the tool predates this standard.

Feature extraction is the processes of identifying useful cell features based on the raw imaging data and summarizing them in a cell-by-feature table (Schapiro *et al.* 2022a). We identify three classes of features: staining-based (e.g. cell mean intensity for a marker), mask-based (e.g. cell area), and spatial context features (e.g. cell centroid coordinates). All cell features are calculated based on the cell segmentation mask, but staining-based features also require the marker images at the cell mask location. Feature extraction methods still often fail to execute on large SP datasets because they require extensive image processing, as each staining-based expression feature is calculated for every marker expressed by every cell. Finding and extracting good features is one of the big challenges in imaging-based spatial omics analysis (Bressan *et al.* 2023).

Another primary analysis task is to cluster cells into distinct cell types or subtypes (Singhal *et al.* 2024). Besides cell clustering, it has been proposed to also look at the imaging data on a pixel level (Liu *et al.* 2022). Pixel clustering can be performed on imaging data without segmentation, allowing for a first image-based quality control step without the need or bias of a deep learning segmentation model. Additionally, the pixel clustering features can be used instead of the image intensity features for a more robust cell phenotyping. While unsupervised pixel clustering is a promising type of analysis and supervised pixel classifiers have been shown to be effective in spatial omics quality control (Martin *et al.* 2024) and are popular in tools such as QuPath (Bankhead *et al.* 2017), such type of analyses are not yet easily accessible from the scverse and SpatialData software ecosystem. Performing it on large datasets directly can be prohibitively expensive, due to the sheer number of pixels and bytes in gigapixel images.

### 1.3 Improved interoperability with existing software ecosystems

Recent analysis standards like the scverse ecosystem for single-cell software tools (Virshup *et al.* 2023), and data storage formats like AnnData (Virshup *et al.* 2021), OME-NGFF (Moore *et al.* 2021), and SpatialData (Marconato *et al.* 2025) aim to improve interoperability between analysis methods. Supporting these standards allows researchers to more easily combine tools to complete their analysis or build reproducible workflows. Tool support for processing microscopy metadata usually is determined by support for the Open Microscopy Environment Compliant specification (Leigh *et al.* 2017) and support for files such as OME-TIFF. While additional metadata has been reported to be useful for quality control (Vierdag and Saka 2024) and there are standardization efforts such as MITI (Schapiro *et al.* 2022b), no current tools have implemented such standards fully. Consistent handling of OME metadata during analysis of multiplexed fluorescence microscopy in new spatial single-cell tools is lacking, compared to mature tools with extensive compliance such as OMERO (Allan *et al.* 2012) and QuPath (Bankhead *et al.* 2017).

## 2 Materials and methods

**Figure 1:**
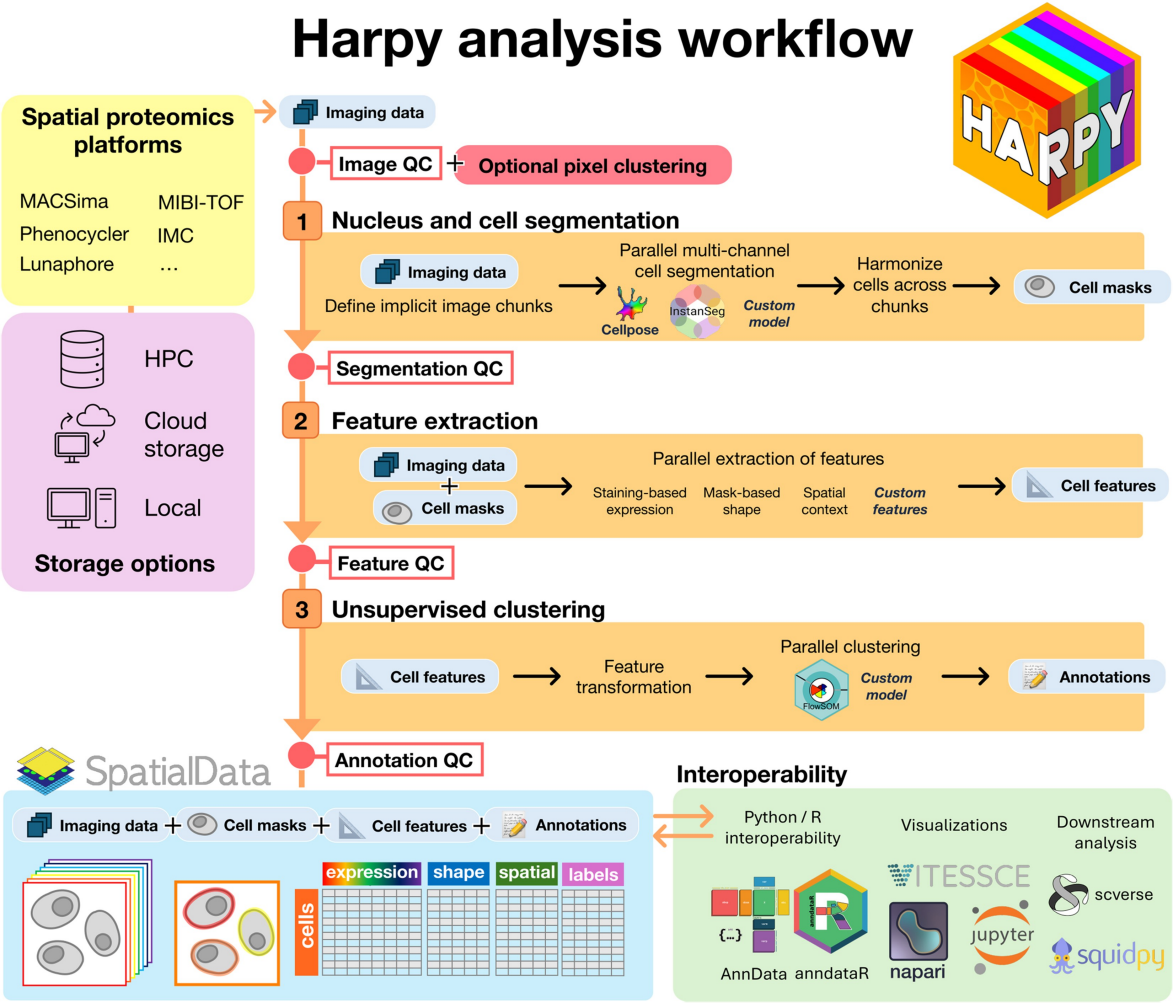
Overview of the Harpy analysis workflow for spatial proteomics data. Imaging data from various acquisition platforms can be read from storage as a SpatialData object. This object can then be extended in three processing steps with cell masks, cell features and cell annotations. Harpy enables visualization and quality control for the output of each of these processing steps. The result is an annotated SpatialData object of single cells with spatial context. Through interoperability support, the resulting data can be read in other programming languages, further visualized or be used in downstream analysis.

The Harpy Python workflow for whole slide spatial proteomics image analysis is summarized in Fig. 1. Data is read from local files, with additional support for remote object storage and high-performance computing (HPC) environments. Spatial proteomics datasets are imported using the spatialdata-io library (Marconato *et al.* 2025), which supports various spatial proteomics platforms, including a MACSima data reader contributed by us. Quality control and image inspection are integrated before and after each processing step, utilizing multiple metrics and plots to summarize large samples in the dataset.

We enable scalable whole slide image processing through the Dask parallel and distributed computing framework (Rocklin 2015), providing Cellpose (Stringer and Pachitariu 2025) and InstanSeg (Goldsborough *et al.* 2024b) as primary segmentation options. Using the cellular or nuclear masks, three different types of features can be calculated for every cell and every marker at a gigapixel scale: staining-based expression features, mask-based shape features and spatial context features.

Informative unsupervised clustering is performed using an updated version of FlowSOM for Python (Couckuyt *et al.* 2024) for which we contributed support for pixel clustering by enabling parallel batch processing and visualizing output in image viewers like napari. Our workflow results in a SpatialData object containing the processed input images, nuclear and cellular masks, and linked feature tables as AnnData objects (Virshup *et al.* 2021). The resulting tables can be read using other tools in the scverse software ecosystem (Virshup *et al.* 2023) such as Squidpy (Palla *et al.* 2022) for further downstream analysis. With the anndataR package (Cannoodt *et al.* 2025), analysis using R packages such as Seurat is possible. Harpy also features interactive and lazy viewing of remote large whole slide images via a diverse set of interfaces, such as Jupyter notebooks, the desktop-based napari software (Sofroniew *et al.* 2022) and the web-based Vitessce framework. Additionally, an analysis feature overview is given in Supplementary S1, comparing Harpy to eight previously reported workflows.

### 2.1 Datasets

We analyzed three publicly available spatial proteomics datasets from human tissue samples: the Liu_2022 Pixie dataset containing 11 MIBI-TOF region-of-interest (ROI) (Liu *et al.* 2022), an IMMUcan_2021 dataset containing 14 IMC samples (Windhager *et al.* 2023) and the Guilliams_2022 Liver Cell Atlas dataset containing 4 MACSima samples (Guilliams *et al.* 2022). We also contribute a new dataset (Tonsil_2022) containing three MACSima ROIs of a human tonsil sample. Characteristics of the datasets can be found in Table 1. All datasets were converted from the published files to a SpatialData Zarr object with processing code available in Harpy.

**Table 1:** Overview of datasets and their characteristics.

Dataset	Tissue	Modality	Samples	Markers	Avg. gigapixels (10^9^)/sample	Avg. cells/sample
Liu_2022 (Liu *et al.* 2022)	Human cancer tissues	MIBI-TOF	11	22	0.021	1394
IMMUcan_2021 (Windhager *et al.* 2023)	Human cancer tissues	IMC	14	40	0.014	3419
Tonsil_2022	Human tonsil	MACSima	3	20	0.886	11 762
Guilliams_2022 (Guilliams *et al.* 2022)	Human liver	MACSima	4	102	3.820	9517

To compare the size and scale, we indicate the number of gigapixels and cells to indicate dataset size. For example, 10^8^ pixels is the size of a medium region-of-interest of 5000 by 5000 pixels and 4 markers. 10^9^ pixels coincides with one gigapixel image sample of 10 markers and 10 000 by 10 000 pixels.

One sample was used for evaluating computational gigapixel scale performance for the tasks of segmentation, feature extraction and clustering: ROI1 of the Tonsil_2022 dataset, with 20 markers (of which two are nuclear stains) and x and y dimensions (7794, 9437). Various dataset sizes were created to evaluate computational performance when pixel sizes increase, all stored as Zarr folders with image chunks of size (c = 1, x = 4096, y = 4096). To obtain smaller total pixel sizes, the original dataset was subsetted in the x and y coordinates. To obtain the larger total pixel sizes, the image ROI was extended beyond 10^8^ pixels via tiled repetition of x and y to simulate a larger sample.

### 2.2 Quality control and visual inspection

SP processing tools should provide visual inspection, multi-sample quality control plots and metadata support before and after all steps of analysis. Harpy provides functions and plots for four levels of quality control, depending on the analysis step output: image level, segmentation level, feature level and annotation level. This functionality is explained through various notebooks on the documentation site for all four mentioned datasets. The quality control levels also correlate with the data levels described in MCMICRO (Schapiro *et al.* 2022a) and quality control levels in IMC Data analysis (Windhager *et al.* 2023). Image level quality control of the marker names is performed by using a supervenn diagram (Fedor 2025). The exact channel names of all different sets are also available in the package. The signal-to-noise ratio plot was implemented as specified by the IMC Data Analysis workflow (Windhager *et al.* 2023).

### 2.3 Scalable processing of images and cells

We performed quantitative comparisons between the Harpy workflow to existing implementations for the tasks of cell segmentation, feature extraction and pixel clustering. For the segmentation task, Slurm jobs were submitted with a wall clock time limit of 24 hours and a memory limit of 128 GiB. For the other two tasks, the limit was 4 hours and 100 GB. Each job was submitted three times, visualized by a mean of the successful jobs with error bars or a confidence interval indicating the range of two standard deviations from this mean, covering 95% of the data when assuming a normal distribution.

For the segmentation task, we compared the Harpy workflow to Sopa and Squidpy using the InstanSeg model (Goldsborough *et al.* 2024b) with default parameters on a 10 channel image and pixel size 0.17. We also performed the same analysis with the functions provided by the InstanSeg package itself. When available, the segmentation chunk size was set to 1000 and the overlap between chunks to 50. The intersection-of-union region at the chunk edge was determined by a pixel region of 2 pixels and the threshold was set at 0.7. A general condition for processing chunks with overlapping borders is that the overlap size should be larger than any cell at the chunk borders. The largest cell area in the dataset was 3044 pixels^2^, which would correspond to a circle with a radius of 31 pixels, making a chunk overlap of 50 pixels sufficient. We varied the number of single-threaded workers and size of the input data. Each method implementation was allowed 1, 4, 8, 16, 32, or 64 workers where possible, and model execution was performed on a CPU. When just using the InstanSeg model with a framework, we set batch_size to 1, as larger batch sizes did not valuably increase runtime while greatly increasing memory usage. For details on the Harpy parallel segmentation algorithm, see Supplementary S2. To show the impact of different parallel segmentation algorithms, we performed a segmentation with the Cellpose cyto3 model on the fov0 sample of the Liu_2022 dataset. We used the default parameters with diameter 30, min_size 10, cellprob_threshold 0 and flow_threshold 0.6. The two input channels are a nuclear (Histone H3) and membrane marker (CD45). The 512 × 512 image was divided in nine chunks of 212 × 212 with an overlap of 50 and reflection at the edges. For Fig. 3, we used the Aggregated Jaccard Index (Kumar *et al.* 2017) to compare the four segmentation examples (E-H) to the reference segmentation (C).

For the feature extraction task in Supplementary S4, we compared Harpy to Squidpy, Sopa, SpatialData and xarray-spatial on calculating the mean expression of each cell across the 20 markers. We used default parameters and varied the number of single-threaded workers and size of the input data. For input segmentation masks, we used the Harpy output of the InstanSeg model created in the segmentation task with eight workers. SpatialData uses xarray-spatial to perform feature extraction, so similar performance is expected. Sopa performs feature extraction on segmentation polygons. In the comparison we ensure the polygons are precalculated and not part of the comparison.

For the clustering task, we compared pixel clustering in Harpy using three different classifier models: the FlowSOM in Python implementation with a regular SOM model (Couckuyt *et al.* 2024), BatchFlowSOM, our new parallel FlowSOM in Python implementation using a batched SOM algorithm, and an alternative Python implementation of the FlowSOM algorithm used in ark-analysis, pyFlowSOM (Liu *et al.* 2022). We used default parameters, a 10 × 10 SOM grid and a data chunk size of 2048, while varying the number of single-threaded workers and size of the input data. For evaluation, the Liu_2022 dataset was used, subsetted for the labeled immune cells and a subset of markers. The unsupervised model was five times fitted on this subset of markers and evaluated on the reference labels using the V-measure, the harmonic mean between cluster homogeneity and completeness. For more details on the input data, marker expression and confusion matrix, see Supplementary S3.

### 2.4 Improved interoperability with existing software ecosystems

Harpy ensure integration with both Python and R-based spatial omics analysis workflows by adopting the standardized SpatialData format. The format is compatible with the scverse ecosystem, enabling downstream analysis using tools such as Squidpy and Scanpy. To support R-based workflows, Harpy leverages new software tools such as SpatialData in R (Crowell 2025) and anndataR (Cannoodt *et al.* 2025).

## 3 Results

We performed various quantitative comparisons between the Harpy workflow to existing implementations for the tasks of cell segmentation, feature extraction and pixel clustering, which are discussed in the next sections. We also discuss our contributions in quality control and interoperability for spatial proteomics data.

### 3.1 Quality control and inspection are facilitated with Harpy

**Figure 2:**
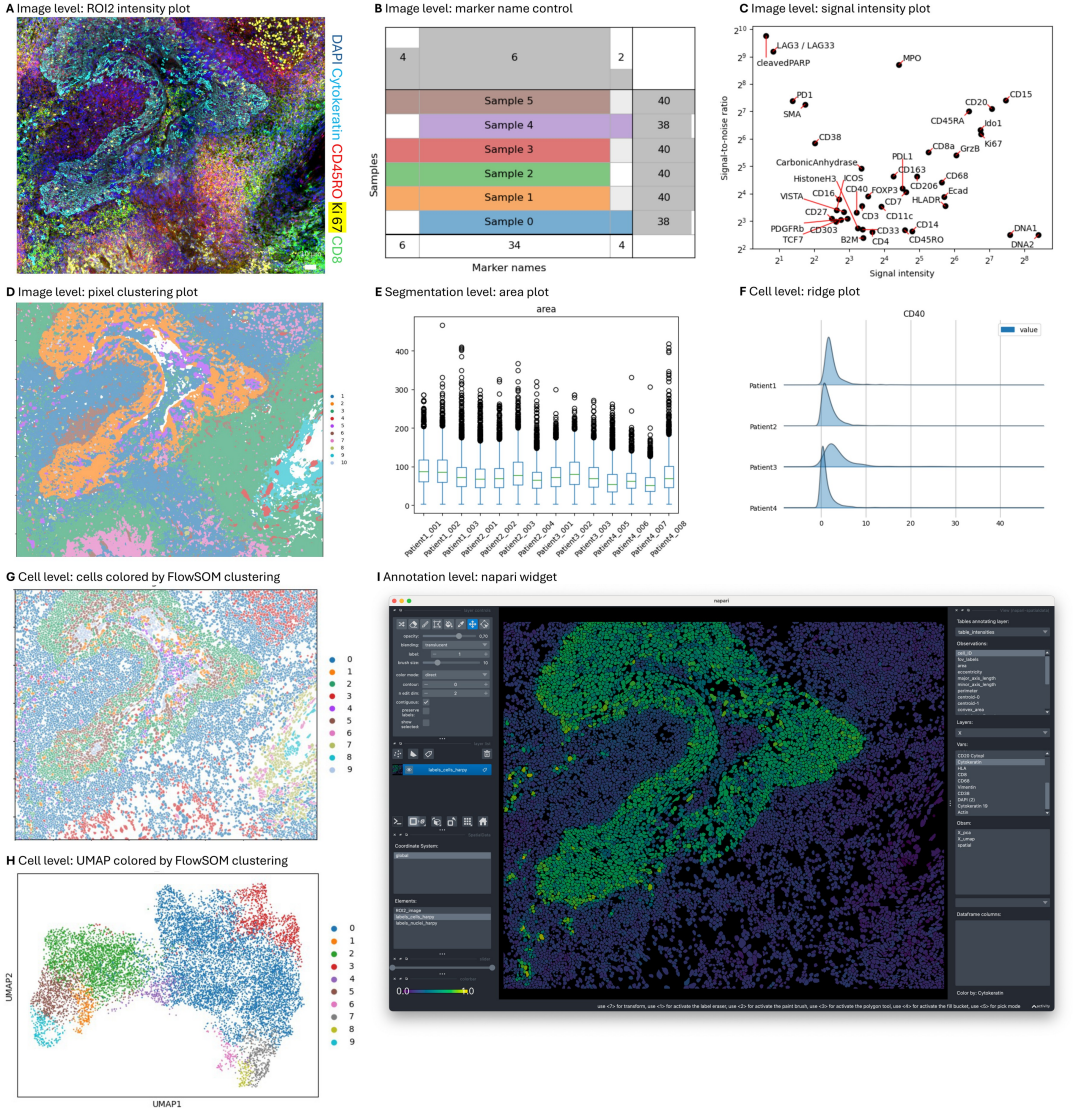
Harpy quality control at various analysis steps. (A) Image plot of intensity values for 5 markers. (B) Image level quality control of consistency of marker names across samples. (C) Scatterplot of the marker signal-to-noise ratio across samples. (D) Image plot of the pixel clustering. (E) Distribution of area of cell masks across multiple samples. (F) Distributions of cell expression for CD40 across sample metadata such as a patient identifier. (G) Cell segmentation mask colored by FlowSOM clustering. (H) UMAP of segmented cells based on mean marker expression, colored by FlowSOM clustering. (I) Screenshot of the Harpy output results, visualized in napari. A, D, G, H, I: sample ROI2 of Tonsil_2022; B: samples of an in-house unprocessed dataset for illustrative purposes; C, E, F: all samples of Immucan_2022.

Quality control in Harpy is organized in four different levels: image, segmentation, cell and annotation. Image level quality control can start after converting the data files from the acquisition platform to the SpatialData format. A notable feature is that Harpy uses SpatialData as a multi-sample container, allowing for quality control across different samples. The unprocessed images can be visualized in Fig. 2 (A), checked for consistency of all marker names across all images in the dataset (B) and dataset-wide metrics such as signal-to-noise for each marker can be plotted (C). Additionally, pixel clustering can be performed to summarize the intensity values of all chosen markers to one overview image (D). For quality control at the segmentation level, Harpy allows visual inspection of the segmentation masks and dataset-wide plotting of mask-based features such as area size (E). At the cell level, Harpy support plotting the distribution of cell intensity for each marker using ridge plots (F) and dimensional embeddings such as UMAP (McInnes *et al.* 2020) (H). At the annotation level, Harpy can show cells in-situ colored by the model output label (G), various plots such as heatmaps and dotplots and allows for visualization in napari for interactive viewing and annotation (I).

### 3.2 Accurate parallel acceleration for segmentation models

**Figure 3:**
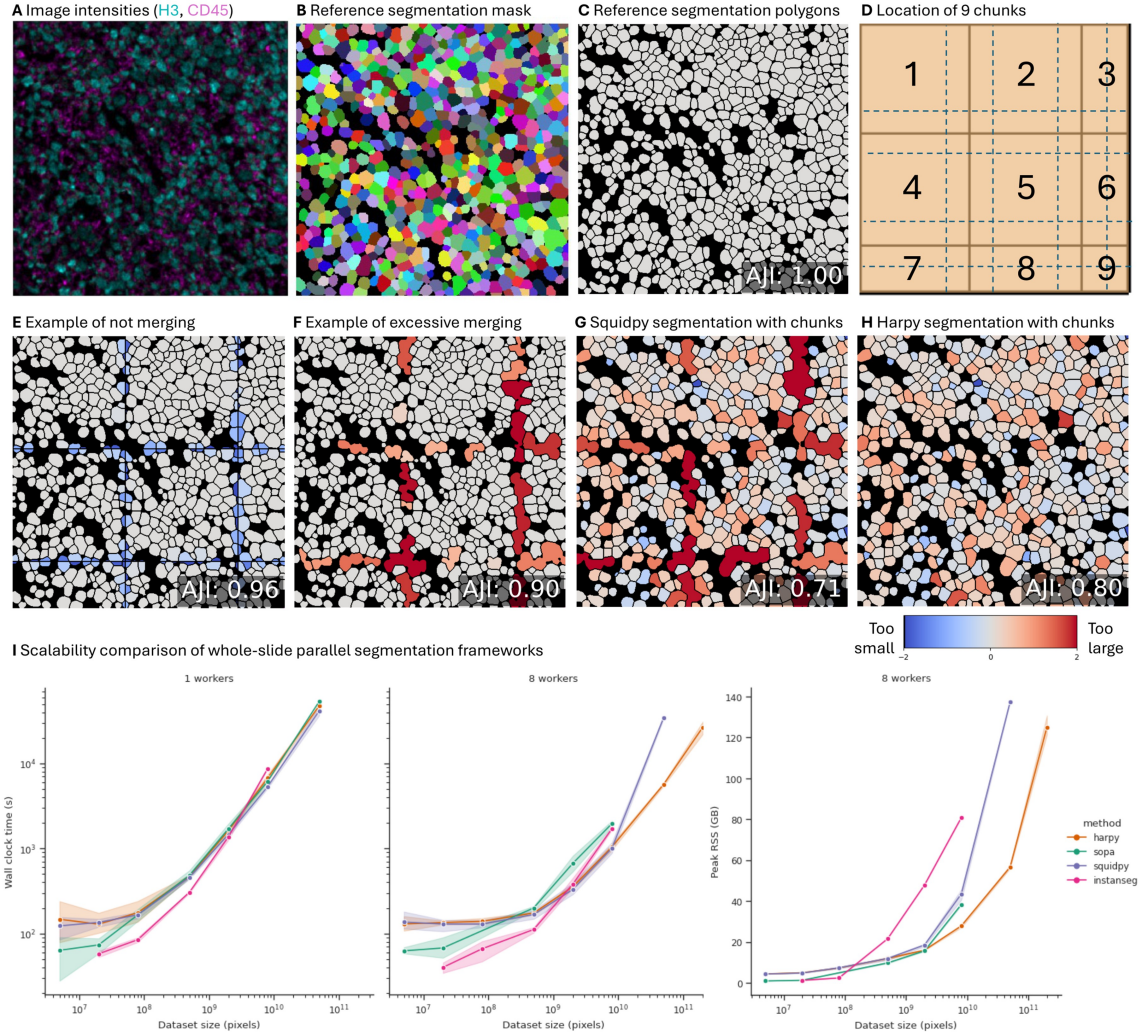
Comparison of whole slide segmentation workflows and impact of parallel acceleration. (A) Image intensities of a nuclear (Histone H3, cyan) and membrane marker (CD45, magenta) of the Liu_2022 MIBI-TOF dataset sample fov0. (B) Ground truth instance segmentation label mask provided with the dataset, each label id colored randomly. (C) Ground truth instance segmentation polygon mask provided with the dataset, each label id colored grey with a 2-pixel dark inner border. Importantly, the masks of the reference labels and the segmentation model output touch without background pixels to separate them. (D) Location of the image chunks created for parallel processing of the sample. The 512 × 512 image is divided in 9 chunks with maximum size of 212x212. An overlap of 50 pixels is indicated with dotted lines. The chunks at the edges are extended by overlap with reflection. (E) Example of technical artefact of not merging the output segmentation at the chunk borders. Predicted segmentations for each cell are colored by the log_2_ of the ratio between the predicted area and ground-truth area. Predicted cells that are too large are red, while predicted cells that are too small are blue (Greenwald *et al*. 2021). (F) Example of technical artefact of excessive Squidpy merging of the output segmentation at the chunk borders. (G) Cellpose default segmentation output using the Squidpy package. (H) Cellpose default segmentation output using the Harpy package. (I) Comparison of the segmentation task using the InstanSeg model using the workflows Harpy, Sopa, Squidpy and InstanSeg itself. More details in Supplementary S2.

We compare whole slide segmentation with and without chunk-based acceleration in Fig. 3. Depending on segmentation mask merging choices at the chunk borders, this can be error-prone. Segmentation masks can be too small when not merging masks at the chunk borders (E). However, segmentation masks at the chunk borders can also be too big when excessively merging when segmentation output masks touch, which might occur in the case of densely populated tissues (F). Importantly, this error can occur silently when using any segmentation model with workflows like Squidpy (Palla *et al.* 2022) (G). One solution is eroding model output masks by one pixel at each chunk, so masks no longer touch, and merging the connecting components. But this would require modifying segmentation model output and does not allow expressing touching masks. Another solution is using an intersection-over-union overlap at the overlapping regions to determine which cell to keep. This can be calculated at the polygon level with workflows like Sopa (Blampey *et al.* 2024), but this involves creating and processing additional polygon masks. The solution in Harpy (H) is to merge overlapping segmentation masks after applying any model in parallel. We show that this is efficient for large datasets and free from the chunked artifact caused by the Squidpy merging (G).

The Harpy workflow can be used to apply different segmentation models, including Cellpose and newer models such as InstanSeg (Goldsborough *et al.* 2024b) with a channel invariant architecture (Goldsborough *et al.* 2024a) that fully use multiple whole slide marker images. When comparing the segmentation time and memory usage of Harpy with other workflows, we find that our parallel segmentation method is scalable for whole slide images. When using only 1 worker, the performance of Harpy is similar to Squidpy, but the implementation does not have the excessive merging behavior. When processing larger datasets, Harpy outperforms InstanSeg and Sopa.

### 3.3 State-of-the-art gigapixel feature extraction

Harpy can extract a diverse set of features from large amounts of imaging data. One challenge in this task is the extensive image processing required, as each staining-based expression feature is calculated for every marker expressed by every cell. We compare our feature extraction implementation to other workflows in Supplementary S4. Above the gigapixel scale, Harpy exceeds the state-of-the art in runtime speed and memory efficiency. When using a time limit of 4 hours and a memory limit of 100 Gigabytes, Harpy succeeded in the extraction task for the largest dataset while other methods were not able to produce results.

### 3.4 Harpy enables fast unsupervised pixel clustering

To assess the speed and memory usage of the implementation, we compare FlowSOM in Python with and without batching and pyFlowSOM. The runtime usage is shown for increasing dataset sizes in Supplementary S5. For images larger than 0.5 gigapixels, the batch training approach shows a faster runtime. With 8 threads and the batch algorithm, FlowSOM in Python is up to 27 times faster on gigapixel images than pyFlowSOM while consuming a similar amount of memory. The batch implementation scales with additional CPU threads and can handle an increasing number of pixels per second, without substantial increase in memory usage.

To assess the quality of unsupervised clustering, the labels of the Liu_2022 dataset are used as reference. The default cluster model of Harpy, FlowSOM in Python, is compared against the cluster model used for Liu_2022, pyFlowSOM. Both models output unsupervised cluster labels, but the reference labels were created with additional manual annotation. The comparable V-measure performance of the different models across different samples indicates that a speed up with batching does not lead to a loss in performance. For fov8, we compare the cluster results visually in-situ and with a UMAP (Supplementary S5).

### 3.5 Harpy interoperates with the single cell software ecosystem

Harpy’s output is designed for broad compatibility with the single-cell and spatial omics software ecosystem. The final result of the workflow is a SpatialData object containing the processed images, nuclear and cellular segmentation masks and feature tables as AnnData objects (Virshup *et al.* 2021). These standardized outputs can be directly used in R and Python-based tools, allowing users to perform further analysis using popular packages such as Squidpy and Seurat without error-prone manual data conversion. Harpy also features interactive and lazy viewing of remote large whole slide images via a diverse set of interfaces, such as Jupyter notebooks, the dektop-based napari software (Sofroniew *et al.* 2022) and the web-based Vitessce framework (Keller *et al.* 2025).

## 4 Discussion and conclusion

In this work, we presented Harpy, a scalable and interactive workflow for the analysis of gigapixel spatial proteomics data. Harpy addresses four critical challenges in the analysis of highly multiplexed bioimaging data: quality control, scalable image processing, scalable clustering, and interoperability. We implement quality control measures and multi-sample plots at multiple levels of analysis, including image, segmentation, feature, and annotation levels.

Harpy leverages the Dask parallel and distributed computing framework to enable efficient processing of large whole slide images. This approach mitigates the limitations of memory and computational resources typically associated with gigapixel images. Additionally, Harpy supports remote datasets and high-performance computing environments, ensuring scalability and security for large-scale analyses. In our comparison, Harpy’s parallel segmentation acceleration is comparative to the state-of-the-art, contains less chunked segmentation artefacts and has reduced processing time and memory usage compared to default application of the segmentation models. If time or computational resources are limited or when comparing a variety of segmentation models like Cellpose and InstanSeg, the scalable parallel segmentation provided by Harpy is particularly interesting. While GPU-accelerated segmentation is also supported, optimal orchestration of such accelerated pre-processing, inference and post-processing for large datasets and various models is a complex matter and still an interest for future research. We show that Harpy allows scalable extraction of a wide array of features and enables processing of such large datasets. Harpy has state-of-the-art runtime and memory usage, allowing it to process larger datasets than other methods. Other methods fail around the gigapixel scale because of two reasons. Methods such as xarray_spatial and SpatialData go over the time limit due to slow processing of all the separate instance segmentation classes. Methods like Squidpy and Sopa require much more memory, slowing them down when applied at gigapixel scale. Harpy’s batch implementation of FlowSOM for Python facilitates pixel clustering and parallel batch processing, reducing computational runtime without a substantial increase in memory usage. When compared to pyFlowSOM on a gigapixel dataset, we achieve a 12× decrease in runtime when single-threaded, and a 27× decrease in runtime when multi-threaded with 8 threads. In our experiments, parallelism becomes effective above data sizes of 10^8^ pixels.

Among currently available tools, only Sopa (Blampey *et al.* 2024) offers comparable integration with the SpatialData standard, having been developed after its release. In contrast, most existing workflows lack standardized mechanisms to link cellular features with their spatial context, limiting their utility for advanced machine learning applications. Harpy addresses this gap by ensuring that all derived features—expression, shape, and spatial context—are stored in a format that is both machine-readable and compatible with widely used analysis frameworks.

Harpy is especially interesting for users who prefer the Python ecosystem, the SpatialData and Dask framework or require large-scale spatial proteomics processing. By accelerating image processing and extracting features at the gigapixel scale, handling and understanding of these promising datasets is improved. The interoperability of Harpy enables the integration of large-scale spatial proteomics processing with various other workflows, including those developed for the analysis of spatial transcriptomics data, paving the way for future multi-modal workflows.

## Supplementary Material

btag122_Supplementary_Data

## Data Availability

The datasets were gathered from sources in the public domain with code available at https://github.com/saeyslab/harpy and archived at https://doi.org/10.5281/zenodo.15546704. Code to reproduce the figures in this work are available at https://github.com/saeyslab/Harpy_figures.
