# Supplementary material for: Scalable analysis of whole slide spatial proteomics with Harpy
Source: Bioinformatics. 2026 Mar 13;42(3):btag122. doi: 10.1093/bioinformatics/btag122 (PMC13064853; doi:10.1093/bioinformatics/btag122)
Supplement: btag122_Supplementary_Data [file btag122_supplementary_data.pdf]

Supplementary

Contents

S1 Workflow feature comparison **2**

S2 Details on parallel instance segmentation **3**

S3 Details on evaluation of clustering task **4**

S4 Performance evaluation of feature extraction task **6**

S5 Performance evaluation of clustering task **7**

S1 Workflow feature comparison

**Workflow**	**Scalability**					**Quality Control**			**Interoperability**		
	Batch processing	HPC support	Parallel WSI	GPU support	Cloud storage	QC across samples	Cyclical metadata	Pixel clustering	Interactive annotation	SpatialData support	Language
QuPath (Bankhead et al. 2017)	Yes		Yes	Yes	Yes			Yes	Yes	Yes	Java
Giotto (Dries et al. 2021)	Yes								Yes		R
Ark-analysis (Liu et al. 2022)	Yes					Yes		Yes	Yes		Python
MCMICRO (Schapiro et al. 2022a)	Yes	Yes		Yes	Yes	Yes					Nextflow
Squidpy (Palla et al. 2022)			Yes	Yes	Yes				Yes	Yes	Python
Steinbock (Windhager et al. 2023)	Yes	Yes		Yes	Yes	Yes			Yes	Yes	Python / R
Sopa (Blampey et al. 2024)	Yes	Yes	Yes	Yes	Yes				Yes	Yes	Python
Giotto Suite (Chen et al. 2025)	Yes		Yes	Yes	Yes	Yes			Yes	Yes	R
SPACEc (Tan et al. 2025)	Yes		Yes	Yes		Yes			Yes		Python
Harpy (ours)	Yes	Yes	Yes	Yes	Yes	Yes	Yes	Yes	Yes	Yes	Python

Table 1: Comparison between Harpy and nine existing analysis workflows for spatial proteomics. We assessed scalability based on support for processing multiple samples in batch or in the same data object, documentation and support for running on high-performance computing (HPC) infrastructure e.g. installation and documentation on job scheduler submission or multi-node environments, documented support for acceleration via a GPU, and documented support for cloud storage e.g. Zarr or S3. For quality control, we assessed the support and documentation for quality control across multiple samples, the usage and support of cycle metadata for multi-cycle highly multiplexed spatial proteomics and the support of pixel clustering. For interoperability, we assessed the support of interactive annotation, readily available support for interacting with the SpatialData format, and the used programming language.

S2 Details on parallel instance segmentation

Algorithm 1 Parallel Instance Segmentation

1: **Input:** Image I, Chunk size C, Overlap size O, IoU threshold T, IoU depth D
2: **Output:** Segmentation mask M
3: Divide I into overlapping chunks $I_1, I_2, \ldots, I_n$ of size C with overlap O
4: **for** each chunk I_i **do**
5: $M_i \leftarrow \text{Segment}(I_i)$
6: $T_i \leftarrow$ Trim chunk M_i by (O - D)
7: $L_i \leftarrow$ Merge connecting labels in adjacent blocks if their IoU $> T$
8: $M_i \leftarrow$ Trim chunk L_i by D
9: **end for**
10: Concatenate all chunks M_i into the final mask M
11: **return** M

		0.005 Gigapixels		0.020 Gigapixels		0.080 Gigapixels		0.500 Gigapixels		2.000 Gigapixels		8.000 Gigapixels		50.000 Gigapixels		200.000 Gigapixels	
		s	GB	s	GB	s	GB	s	GB	s	GB	s	GB	s	GB	s	GB
harpy	1	123	1	111	2	151	2	476	4	1578	6	6666	16	48189	43	-	-
	2	150	2	140	2	143	3	296	5	911	7	3059	17	22548	44	-	-
	4	179	3	118	3	125	5	193	7	506	10	1703	20	10719	47	51204	**107**
	8	122	4	135	5	138	7	178	11	356	15	1036	26	5615	52	26577	114
	16	106	7	176	8	155	9	157	17	251	25	673	37	3239	63	12802	127
	32	139	13	132	13	128	15	142	26	176	38	403	57	1769	84	**7487**	127
	64	130	24	125	24	150	26	136	39	**157**	65	285	93	**1087**	133	10708	133
sopa	1	72	**1**	75	1	-	-	453	**2**	1652	**2**	6102	**3**	53512	**20**	-	-
	2	80	1	65	1	-	-	316	4	1257	9	6670	29	-	-	-	-
	4	57	1	58	1	-	-	214	6	628	11	3346	28	-	-	-	-
	8	61	1	62	1	-	-	204	9	652	14	1958	36	-	-	-	-
	16	**53**	1	84	1	-	-	158	15	297	21	1011	41	-	-	-	-
	32	60	1	60	1	-	-	130	25	226	32	695	52	-	-	-	-
	64	63	1	64	1	-	-	124	38	180	57	371	82	-	-	-	-
squidpy	1	128	1	138	2	169	2	450	4	1500	10	5428	33	40907	90	-	-
	2	131	2	135	2	171	3	262	5	840	11	2938	35	36225	127	-	-
	4	126	2	100	3	116	5	202	8	479	12	1515	37	41367	128	-	-
	8	115	4	138	4	125	7	166	11	310	17	947	41	33952	128	-	-
	16	140	7	153	7	120	10	151	20	221	27	582	50	-	-	-	-
	32	121	13	132	13	144	16	116	30	196	42	420	71	31658	128	-	-
	64	140	24	145	24	149	26	130	43	180	72	**260**	103	31340	131	-	-
instanseg	1	-	-	58	1	85	3	305	8	1398	18	8645	67	-	-	-	-
	2	-	-	52	1	45	3	201	12	785	19	4796	67	-	-	-	-
	4	-	-	**34**	1	46	2	151	20	571	28	3121	68	-	-	-	-
	8	-	-	40	**1**	71	2	115	20	346	44	1680	75	-	-	-	-
	16	-	-	48	1	51	2	82	18	283	78	1164	110	-	-	-	-
	32	-	-	43	1	49	**2**	81	18	191	78	-	-	-	-	-	-
	64	-	-	53	1	**36**	2	**67**	18	176	79	-	-	-	-	-	-

Table 2: Details for segmentation comparison of Figure 3. Four methods were compared on 8 increasing levels of dataset size for 7 increasing level of maximum amount of workers. The median values of three repeated executions is shown for two metrics: execution time (seconds) and maximum memory usage (GB). Three regions of missing values are present: 1) For Sopa, the 0.080 gigapixel dataset failed because of the InstanSeg error ("Reached maximum number of iterations - this is not expected!"). 2) InstanSeg failed to execute on the smallest dataset size due to an error for eval_medium_image, as the overlap was smaller than the window size. The other models make use of the InstanSeg function eval_small_image, which works but does not support parallel segmentation and chunk merging logic. 3) Missing values at the right side of the table indicate execution time or memory issues due to the large dataset. Note that the time limit is 24 hours and the memory limit of 128 GiB translates to around 137 GB. Note that Sopa and InstanSeg avoid copying the data to multiple workers for lower sizes, reducing the memory usage.

S3 Details on evaluation of clustering task

The Liu_2022 dataset has 11 field-of-views with 22 marker channels. In bold are the 9 selected immune markers for clustering: **CD3, CD4, CD8, CD14, CD20**, CD31, **CD45, CD68, CD163**, CK17, Collagen1, ECAD, Fibronectin, GLUT1, H3K9ac, H3K27me3, **HLADR**, IDO, Ki67, PD1, SMA, Vim. The used cell types labels are Bcell, CD14_monocyte, CD4T, CD8T, M1_macrophage and M2_macrophage.

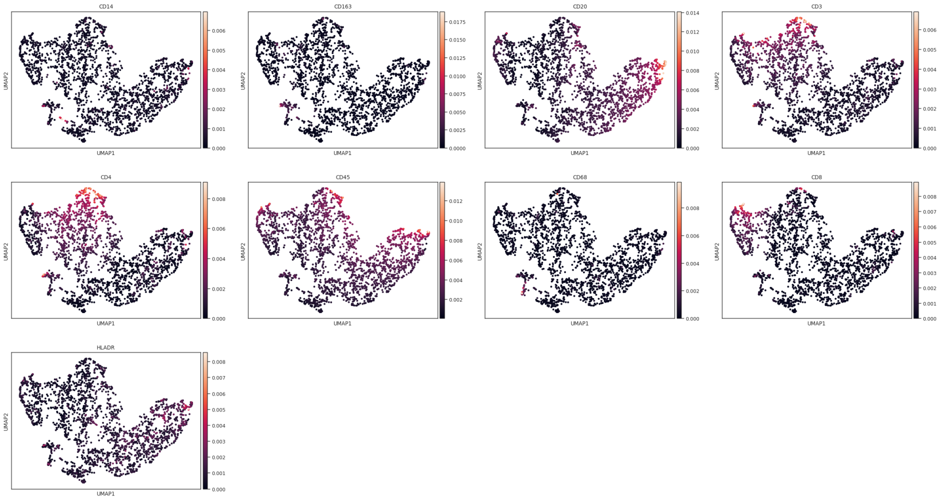

Figure 1: Individual marker expression on UMAP plot for fov8.

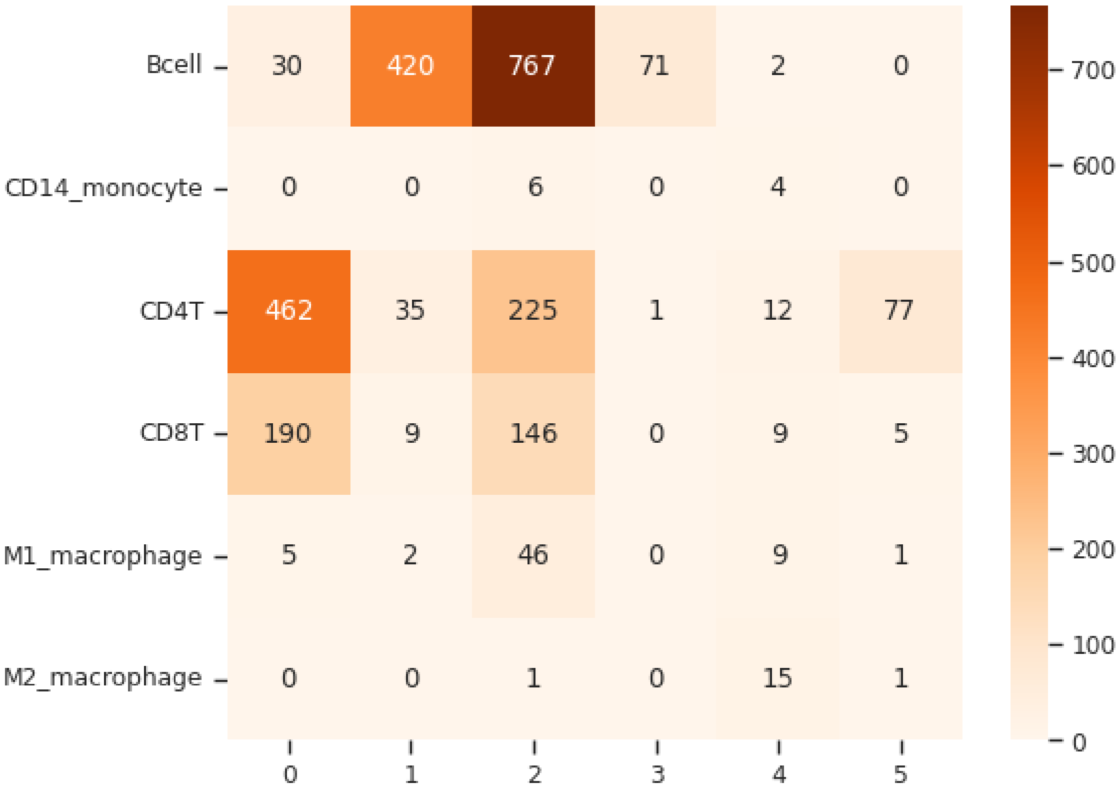

Figure 2: Confusion matrix of the BatchFlowSOM in Python implementation for fov8.

S4 Performance evaluation of feature extraction task

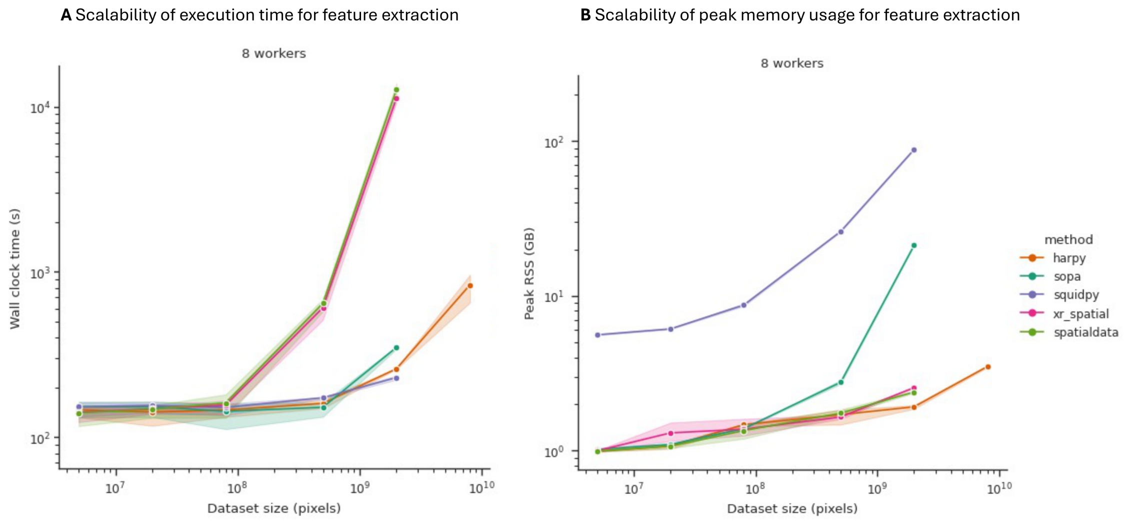

Figure 3: Scalability comparison of feature extraction workflows. (A) Comparison of between workflows when calculating the mean intensity of cells for each marker (B) Comparison between workflows when calculating the mean intensity of cells for each marker.

S5 Performance evaluation of clustering task

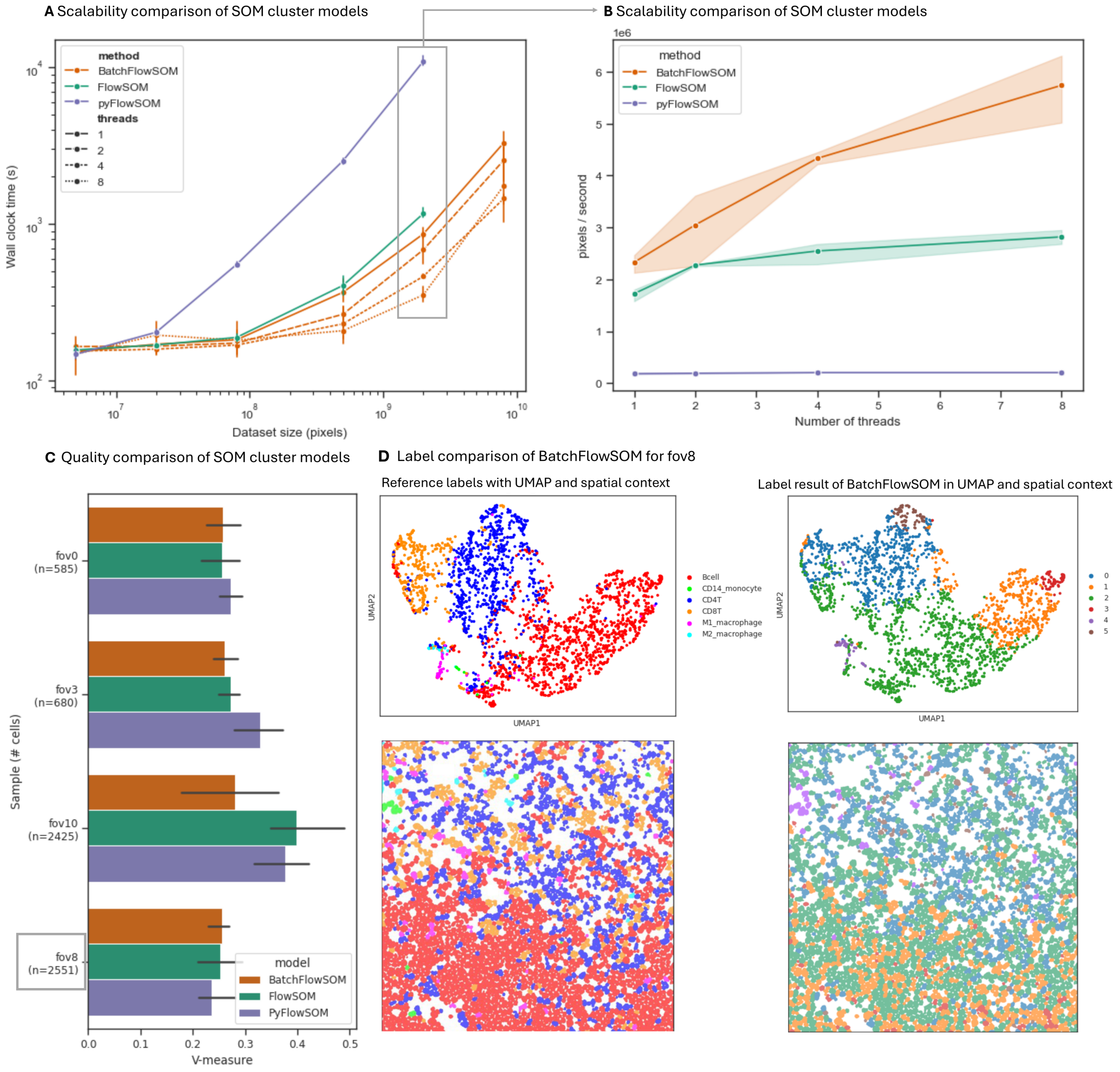

Figure 4: Comparison of Harpy with different pixel clustering models. (A, B) Scalability comparison when performing pixel clustering in Harpy using three different SOM models. As dataset sizes increase, we measure wall clock time (seconds) and scalability when more CPU threads are allowed (pixels processed per second) (C) Quality comparison of unsupervised clustering with as reference labeling the fine-tuned cell clustering provided in the ark-analysis example dataset. Four field-of-views (fov) are analysed, shown on the x-axis, and the V-measure for the three SOM models is shown on the y-axis. (D) The reference labels for fov8 are shown in-situ and in a UMAP. The unsupervised clustering result with BatchFlowSOM on fov8 are also shown in the same UMAP embedding.

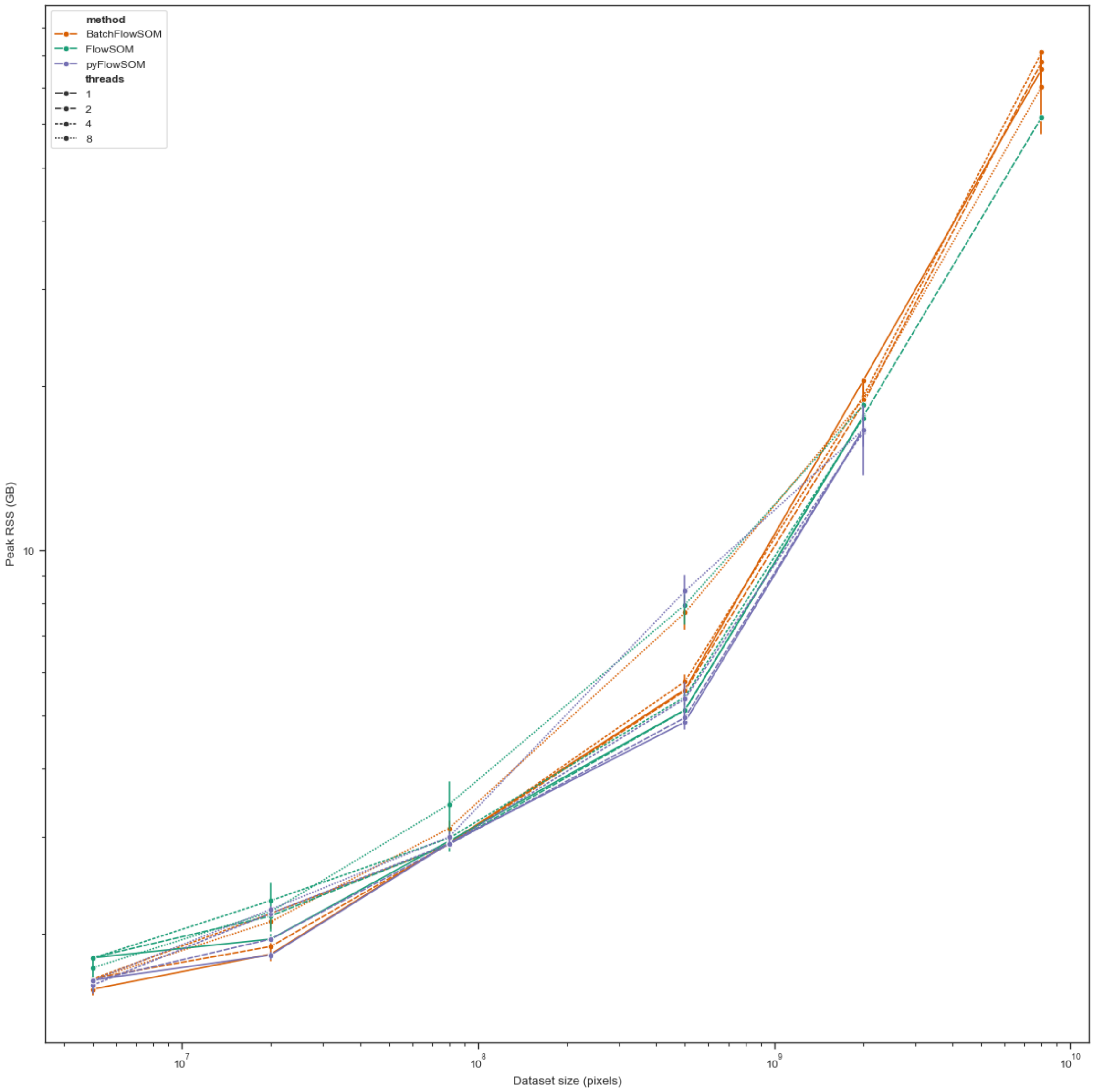

Figure 5: Memory usage of SOM clustering comparison. The memory usage for the $2 * 10^9$ dataset with pyFlowSOM and 1 thread is 16.66 GB. The memory usage with BatchFlowSOM and 8 threads is 18.88 GB.

		0.01 Gigapixels		0.02 Gigapixels		0.08 Gigapixels		0.50 Gigapixels		2.00 Gigapixels		8.00 Gigapixels	
		Time (s)	Memory (GB)	Time (s)	Memory (GB)	Time (s)	Memory (GB)	Time (s)	Memory (GB)	Time (s)	Memory (GB)	Time (s)	Memory (GB)
method	threads												
BatchFlowSOM	1	152.47	**1.58**	170.29	1.84	181.70	2.93	365.29	5.58	862.59	20.44	3293.20	75.57
	2	164.41	1.66	165.36	1.90	173.08	2.92	264.53	5.55	683.31	18.59	2550.44	77.93
	4	153.81	1.65	158.25	2.18	168.09	**2.90**	229.72	5.77	461.96	19.12	**1450.39**	81.15
	8	147.26	1.65	194.57	2.10	180.68	3.11	**207.70**	7.70	**351.63**	18.88	1751.57	70.22
FlowSOM	1	156.44	1.81	168.34	1.96	188.26	2.95	403.92	5.12	1163.67	17.58	-	-
	2	154.99	1.81	163.33	2.15	175.74	2.91	324.65	5.11	879.07	17.58	11227.04	**61.78**
	4	145.62	1.80	**152.86**	2.30	**156.96**	2.99	313.41	5.41	789.36	17.46	-	-
	8	**145.32**	1.73	160.83	2.19	187.01	3.44	280.44	7.96	710.55	18.42	-	-
pyFlowSOM	1	146.83	1.65	203.08	**1.83**	550.62	2.92	2527.57	**4.87**	10942.15	16.66	-	-
	2	163.29	1.64	201.63	1.96	512.18	2.91	2380.64	4.96	10430.77	16.66	-	-
	4	148.60	1.61	189.27	2.18	530.00	2.92	2335.93	5.37	9779.74	**16.45**	-	-
	8	155.04	1.64	229.51	2.21	516.77	3.00	2315.98	8.44	9767.74	16.62	-	-

Table 3: Details for clustering task comparison of S5. The best performers or lowest values per outcome metric are indicated in bold. Note that missing values on the right side are indicative of execution time or memory issues due to the dataset size.
